# The effect of interventions anticipated to improve plantar intrinsic foot muscle strength on fall-related dynamic function in adults: a systematic review

**DOI:** 10.1186/s13047-021-00509-0

**Published:** 2022-01-20

**Authors:** Lydia Willemse, Eveline J. M. Wouters, Henk M. Bronts, Martijn F. Pisters, Benedicte Vanwanseele

**Affiliations:** 1 Department of Health Innovations and Technology, Fontys University of Applied Sciences, PO Box 347, 5600 AA Eindhoven, The Netherlands; 2 Department of Movement Sciences, KU Leuven, Tervuursevest 101 - box 1500, 3001 Leuven, Belgium; 3 Tranzo, School of Social and Behavioral Sciences, Tilburg University, PO Box 90153, 5000 LE Tilburg, The Netherlands; 4 Department of Rehabilitation, Physiotherapy Science & Sport, UMC Utrecht Brain Center, Utrecht University, PO Box 85500, 3508 GA Utrecht, The Netherlands; 5 Center for Physical Therapy Research and Innovation in Primary Care, Julius Health Care Centers, PO Box 85500, 3508 GA Utrecht, The Netherlands

**Keywords:** Intrinsic foot musculature, Exercise therapy, Balance, Gait, Falling

## Abstract

**Background:**

The plantar intrinsic foot muscles (PIFMs) have a role in dynamic functions, such as balance and propulsion, which are vital to walking. These muscles atrophy in older adults and therefore this population, which is at high risk to falling, may benefit from strengthening these muscles in order to improve or retain their gait performance. Therefore, the aim was to provide insight in the evidence for the effect of interventions anticipated to improve PIFM strength on dynamic balance control and foot function during gait in adults.

**Methods:**

A systematic literature search was performed in five electronic databases. The eligibility of peer-reviewed papers, published between January 1, 2010 and July 8, 2020, reporting controlled trials and pre-post interventional studies was assessed by two reviewers independently. Results from moderate- and high-quality studies were extracted for data synthesis by summarizing the standardized mean differences (SMD). The GRADE approach was used to assess the certainty of evidence.

**Results:**

Screening of 9199 records resulted in the inclusion of 11 articles of which five were included for data synthesis. Included studies were mainly performed in younger populations. Low-certainty evidence revealed the beneficial effect of PIFM strengthening exercises on vertical ground reaction force (SMD: − 0.31-0.37). Very low-certainty evidence showed that PIFM strength training improved the performance on dynamic balance testing (SMD: 0.41–1.43). There was no evidence for the effect of PIFM strengthening exercises on medial longitudinal foot arch kinematics.

**Conclusions:**

This review revealed at best low-certainty evidence that PIFM strengthening exercises improve foot function during gait and very low-certainty evidence for its favorable effect on dynamic balance control. There is a need for high-quality studies that aim to investigate the effect of functional PIFM strengthening exercises in large samples of older adults. The outcome measures should be related to both fall risk and the role of the PIFMs such as propulsive forces and balance during locomotion in addition to PIFM strength measures.

**Supplementary Information:**

The online version contains supplementary material available at 10.1186/s13047-021-00509-0.

## Background

Annually, approximately one third of the adults aged over 65 year fall at least once and this number increases with advancing age [1]. One third of all falls result in serious injury [2]. These injuries eventually lead to hospitalization, institutionalization, or mortality in a substantial number of events [3]. Since the rate of serious injuries as a consequence of a fall in older adults increases over the years and because of its financial and societal consequences, there is an urgent need for improving the prevention of falling in this specific population.

Altered gait and related balance deficits are strong determinants of falling [2, 4, 5] and very common [4, 6, 7] in older adults. A wide range of biological factors can be related to these age-related changes. However, there is rationale to believe that the decreased force-producing capacity of the plantar intrinsic foot muscles (PIFMs), as observed in older adults [8], may have a role in at least two aspects of the functional decline that make them more likely to fall. These comprise of diminished balance control in dynamic circumstances and reduced generation of propulsive power.

Older adults experience difficulties to control their balance during gait, particularly in the frontal plane [9, 10]. Mechanically, this can be explained by the narrowed mediolateral stability margins in comparison with younger adults resulting from increased sway of the body’s center of mass in this direction [9] in conjunction with the more medially directed progression of the center of pressure (CoP) throughout the loading phase of gait in older adults [11]. This medial shift of the CoP has been associated with a lower medial longitudinal foot arch (MLA) [12], which is typical of the aging foot [13, 14]. A flatter foot might also negatively impact dynamic balance as this causes increased motion of the forefoot, reflecting reduced stiffness of the foot [15]. This lack of a high arched stiff foot resulting in a less stable base of support might be due to insufficient force produced by the PIFMs [16–18]. In addition to this role in dynamic balance during gait, the PIFMs also play a role in static postural balance, especially in the frontal plane or when the postural demand of the task is increased [19, 20]. Hence, it is likely that the observed atrophy of the PIFMs in older adults interferes with the capability of the postural system to remain balanced during gait.

Not only decreased control of balance is typical for older adults’ gait, but also reduced generation of propulsive power [7, 21]. A reduced push-off may result in smaller steps [22], slower walking speed [7] and increased stance time [21]. These spatiotemporal gait parameters are all associated with an increased risk of falling [23]. The reduced propulsive power has been previously attributed to the decreased capacity of the ankle plantar flexors to generate power at the ankle joint [22, 24]. However, since the foot is simply modelled as one rigid body in these studies, it remains unclear to which extent the foot contributed to the estimated ankle joint power [25]. Instead, a recent study, using a multi-segment foot model [26], showed that normal push-off was jeopardized when the PIFMs were unable to contract. This implies that effective force transmission to the ground may be hindered by the diminished force capacity of the PIFMs in older adults.

Thus, for older adults, PIFMs that function properly (e.g., have sufficient strength and endurance) seem to be important to walking safely. This is further supported by the finding that toe flexion strength, both credited to the PIFMs and the extrinsic foot muscles [27], is an independent predictor of dynamic balance performance in older adults [28]. Furthermore, older adults with less toe flexion strength were more likely to fall and this variable was more discriminative than a combination of other intrinsic factors, such as proprioception and quadriceps muscle strength [29]. Assuming that a loss of muscle strength is a reversible process in older adults [30], older adults may benefit from strengthening the PIFMs in order to improve or retain their gait performance and decrease the risk of falling.

Despite the potential of strengthening the PIFMs, only a few studies investigated the effect of strengthening exercises for the foot muscles in older adults, with no attempts made to distinguish between the contribution of intrinsic and extrinsic foot muscles. These studies revealed consistent results: increased toe plantar flexion strength [31–33] and improved balance performance [31, 32], while gait speed remained unchanged [32, 33]. Additionally, a systematic review [34] and a randomized controlled trial (RCT) [35] evaluating programs consisting of both foot and ankle exercises, not limited to strengthening exercises, concluded these programs to be beneficial for static balance [34, 35] and reducing the number of falls [35]. However, to the extent of our knowledge, no studies investigated the effect of strength training directed specifically at the PIFMs on fall risk related outcomes in older adults. Therefore, the preventative effect of strengthening the PIFMs in this population at high risk to falling remains unclear. Nevertheless, evidence regarding the effect of such interventions in adults of all ages on parameters associated with a higher fall risk in older adults would enhance our comprehension of its potential in older adults.

A recent (2017) systematic review by Huffer et al. [36] investigated if plantar foot strength training interventions were effective in the treatment or prevention of plantar fasciitis and in improving intrinsic foot muscle strength. Based on primarily healthy study populations not expected to be at increased risk of plantar fasciitis, the authors could not draw a convincing conclusion on the effect of PIFM strength training on functional performance due to the diverging outcome measures used in the included studies. Since this systematic review, numerous studies emerged in which the effect of interventions aimed to improve PIFM strength was investigated. These studies demonstrated a beneficial effect pertaining to PIFM strength and hypertrophy [37–41]. However, it is not clear to what extent this reflects improved dynamic function. As PIFMs are primarily engaged in dynamic functions and these are vital to walking in older adults, insight in the effect of PIFM strengthening interventions on these locomotor functions would be helpful in optimizing fall prevention programs. Therefore, the aim of the current systematic literature review was to provide insight in the evidence for the effect of interventions anticipated to improve PIFM strength on dynamic balance control and foot function during gait in adults.

## Methods

This systematic review has been reported according to the PRISMA statement [42]. The protocol is registered and accessible in the PROSPERO database under the number CRD42020197788.

### Search strategy

PubMed, CINAHL Plus with full text, SPORTSDiscus with full text, PEDRO and Web of Science were used to search the literature for peer-reviewed articles. Because no intervention studies concerning PIFM strength training were expected to be published before 2010 based on the review of Huffer et al. [36], the search was limited to publications between January 1, 2010 and July 8, 2020. The search strategy applied in PubMed is shown in Table 1 and the equivalent strategies for the other databases can be found in Additional file 1. The search string was built from three sets of terms related to 1) the type of intervention, 2) the target of the intervention and 3) outcome measures. A fourth set was added to exclude articles concerning neurological pathologies known to cause gait impairment. Available MeSH terms or subject headings that relate to “strength training” or “exercise therapy” were explored and included in the search string whenever applicable. A library information specialist was involved in establishing the search string. If allowed by the search engine, the search was restricted to full text articles written in English or Dutch, languages that the researchers can read and interpret at a proficient level, and reporting studies on human subjects. Additional records were either found by checking the reference lists of included articles or by forward citation tracking of the same articles using Google Scholar on September 1, 2020. All five databases were checked for relevant articles published afterwards on April 26, 2021. These additional searches were performed by a single investigator (LW).

**Table 1:** Search strategy as applied in PubMed

**Type of intervention:**	AND	“exercise therapy” [MeSH Terms] OR “resistance training” [MeSH Terms] OR exercise OR strengthening OR shoes OR footwear OR barefoot OR foot ortho* OR insole* OR inlay*
**Target of intervention:**	AND	doming OR “short foot” OR “foot core” OR foot musc* OR intrinsic foot OR plantar musc* OR toe musc* OR hallu* muscle
**Outcome measure:**	AND	postur* balance OR postur* stability OR postur* control OR stance balance OR stance stability OR stance control OR dynamic* OR function* OR gait OR walking OR locomotion OR running
**Pathologies:**	NOT	stroke OR “multiple sclerosis” OR “cerebral palsy”

### Selection criteria

Studies were included if characterized by:

- a study **population** consisting of adults of all ages in the absence of a neuromuscular or neurological condition affecting lower extremity function severely and without any painful musculoskeletal complaints in the lower extremity. The latter is expected to interfere with exercise performance and is negatively associated with adherence [43];
- studying the effect of noninvasive **interventions** anticipated to improve PIFM strength. As the focus is on strength, as opposed to neuromuscular adaptations, interventions had to last at least 4 weeks [38, 44]. These interventions include, for example but were not restricted to, 1) muscle strengthening programs composed of ‘short foot’ or ‘foot doming’ exercises or exercises requiring toe flexion muscle force (e.g., toe plantar flexion, towel curl exercise, marble pick up, heel raises) or toe ab−/adduction muscle force (e.g., toe spread out), 2) a transition from conventional to minimal shoe or barefoot condition;
- reporting **outcome measures**, at least assessed at baseline and directly post intervention, that are related to the locomotor system’s function on balance control and propulsion. These measures should originate from the following domains: 1) dynamic balance (e.g., star excursion balance test), 2) foot and ankle biomechanics during gait or running (e.g., MLA kinematics, kinetics, plantar pressure, propulsive power of foot and ankle joints), 3) anterior and vertical ground reaction force (GRF) peak and impulse at push-off phase during gait or running and 4) spatiotemporal gait or running parameters.
  In addition, if the intervention targeted other muscles additional to the PIFMs (e.g., heel raises, foot and ankle exercise program, transition to minimal shoe or barefoot condition), then at least the outcome of one measure of purely PIFM strength (e.g., size or doming strength) should be reported, in order to be able to associate changes in dynamic outcome measures to changes in PIFM strength.
- one of the following **study designs**: 1) a controlled trial in which one of the above mentioned interventions was the contrast between the trial arms (i.e., intervention A compared to intervention A + intervention of interest) or was compared with ‘no intervention’/ ‘placebo’ (e.g. stretching)/ ‘usual care’ (e.g., usual training regime), 2) a controlled trial in which at least one group received only the intervention of interest, but without an adequate control group as described in 1. This was considered as a pre-post interventional study in further analysis, and 3) a pre-post interventional study in which the study population received only the intervention of interest.

Articles were excluded when the intervention was described as 1) an exercise intervention not only focusing on toe, foot or ankle muscles, (e.g., fall prevention programs, rehabilitation therapy), 2) balance or proprioceptive training, 3) running training or walking program without a transition to minimal shoe or barefoot condition, or 4) post-operative therapy.

### Selection process

Subsequent to automatic duplicate removal (Covidence systematic review software, Veritas Health Innovation, Melbourne, Australia. Available at www.covidence.org), the titles and abstracts were screened for possible eligible studies by two reviewers (LW, HB) independently. After reaching consensus, the full text of these remaining records was evaluated according to the selection criteria, by the same reviewers blinded to each other’s decision. Any decisional inconsistency regarding inclusion or the reason for exclusion were resolved through discussion. A third reviewer (BV) was available during the process to resolve any remaining conflict.

### Data extraction

Predefined data sheets were customized to extract sample characteristics, description of intervention, methodology used to assess outcomes, results on outcome measures of interest (including PIFM strength if reported) at baseline and directly post-intervention for each outcome domain and the statistical significance of comparisons (i.e., group x time interaction effect for controlled trials and time effect for pre-post interventional studies). In case an article reported multiple outcomes within the same outcome domain, the outcome measure that is advocated by the literature as most closely related to the role of the PIFMs was selected for further synthesis [45, 46]. If equally related, further decisions were first made based on the availability of data, second on statistical significance and last on the effect size. Extraction was performed by a single reviewer (LW) and checked for correctness by the other reviewer (HB). The data were tabulated for presentation purposes.

### Methodological quality assessment

The Downs & Black checklist [47] was used to assess the methodological quality of the included articles independently by two reviewers (LW, HB). The checklist contains 27 questions addressing clarity and completeness of reporting (10 items), external validity (3 items), internal validity (13 items) and power (1 item). This checklist has been used previously in systematic reviews and is applicable to evaluate various study designs [36, 48, 49]. Item 23 and 24 are not applicable to non-randomized studies and additional to these items, item 5, 21, 22, 25 do not apply to pre-post interventional studies. These items were therefore scored ‘unable to determine’ in these cases. As such, a pre-post interventional study is inherently classified as being of less quality compared to an RCT. Item 27, concerning the power of the study, was transformed into a dichotomous scale indicating whether or not a sample size calculation was reported [36]. The checklist as it was used in the current review is provided in Additional file 2. In case no consensus was reached on an item, a third reviewer (BV) was available for a final decision. Only the total score on the construct ‘internal validity’ was used to determine the methodological quality of the study. A study with a score on ‘internal validity’ between 0 and 4 was designated as of ‘low quality’, a score between 5 and 8 as of ‘moderate quality’ and between 9 and 13 as of ‘high quality’ [49].

### Data analysis

Effect sizes of comparisons were expressed in standardized mean differences (SMD) and calculated according to the formulas proposed by Lakens et al. [50]. The standardized mean difference between groups in change from baseline for the sample was given by Cohen’s *d*_*s*_:

1 $$ {d}_s=\frac{\overline{X_{I, d\iota ff}}-\overline{X_{C, d\iota ff}}}{\sqrt{\frac{\left({n}_I-1\right)s{d}_I^2+\left({n}_C-1\right)s{d}_C^2}{n_I+{n}_C-2}}} $$

where *C* and *I* indicate the control group and intervention group, respectively. $\overline{X_{I, d\iota ff}}-\overline{X_{C, d\iota ff}}$ is the difference in the change from baseline between the control group and the intervention group. This numerator was preferred over the between-groups post-intervention difference. This was because baseline measures tended to differ between groups and therefore, the between-groups post-intervention difference may not represent an intervention effect adequately. The pooled pre-intervention standard deviation was used as the denominator in Formula 1 as an alternative to the standard deviation of the changes from baseline, because there was not enough information available to determine the latter for the majority of studies. For the same reason, it was impossible to determine the confidence interval of the SMD.

In order to be able to interpret the SMD across study designs (i.e., between-group and within-group designs), the SMD for within group differences was given by Cohen’s *d*_*av*_:

2 $$ {d}_{av}=\frac{M_{t_1-{t}_0}}{\frac{s{d}_{t_0}+{sd}_{t_1}}{2}} $$

where *t*_*0*_ and *t*_*1*_ indicate the baseline and post-intervention measurement, respectively and ${M}_{t_1-{t}_0}$ is the mean change from baseline.

The SMDs (i.e., *d*_*s*_ and *d*_*av*_) were corrected in case of a small sample size (*n* < 20) [50] resulting in Hedges’s *g*_*s*_ and *g*_*av*_:

3 $$ {g}_s={d}_s\times \left(1-\frac{3}{4\left({n}_I+{n}_C\right)-9}\right) $$

4 $$ {g}_{av}={d}_{av}\times \left(1-\frac{3}{4(2n)-9}\right) $$

The SMD values were transformed in a way that positive values indicate an improvement in the outcome measure favoring the intervention. An SMD below 0.5 was interpreted as a small effect, between 0.5 and 0.8 as a moderate effect, and ≥ 0.8 as a large effect [51].

### Data synthesis

A meta-analysis was not undertaken because the unknown variance of the change from baseline impeded adequate calculation of confidence intervals around the effect estimates [52]. In spite of efforts to request the required data from the authors of included articles, only one author provided these data. Therefore, the method of ‘summarizing effect estimates’ was applied instead [52]. Only studies of moderate and high methodological quality were used in the synthesis [52].

The GRADE approach [53] was used to assess the certainty of evidence for each outcome domain for which at least one moderate or high quality study was included and only by taking the moderate and high quality studies into account [52]. Starting with an initial ‘high’ score, the quality of evidence was subsequently downgraded by one or two levels based on concerns on these five factors:

1. Risk of bias
2. Inconsistency of results
3. Indirectness of evidence
4. Imprecision of results
5. Probability of publication bias

The final grade was ‘high’, ‘moderate’, ‘low’ or ‘very low’ and reflects the certainty of the true effect for each outcome domain.

A sensitivity analysis was performed to assess the robustness of the level of evidence by modifying the lower boundary for classifying the studies as being of moderate methodological quality (i.e., ≥ 4 or ≥ 6, rather than ≥5).

## Results

### Study selection

**Fig. 1:**
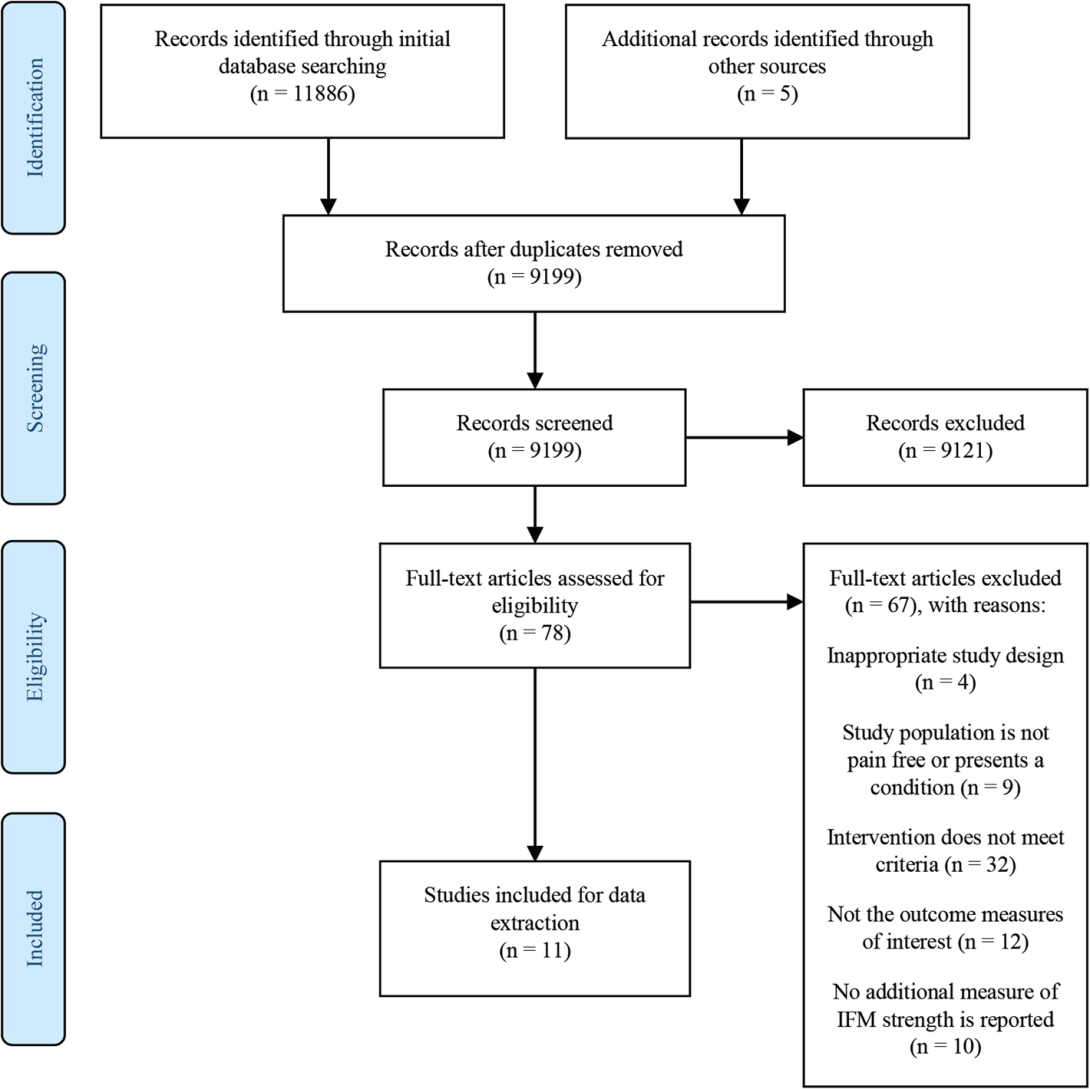
*PRISMA flow chart* [42] *of applied selection process*

The process of study selection is shown in Fig. 1. In the databases and through forward and backward citation tracking, 9198 unique records were identified. One extra article was added as a result of the additional search in the most recent literature. Among these records, 78 articles were deemed relevant based on title and abstract. The screening of the full texts resulted in inclusion of 11 studies that met the selection criteria. Among the excluded studies were the studies of Spink et al. [35] and Okamura et al. [41], because the interventions incorporated more than only toe, foot and ankle strengthening exercises in combination with the fact that isolated intrinsic foot muscle strength or strength capacity was not evaluated.

### Characteristics of included studies

The characteristics of the included studies are summarized in Table 2. These studies consist of four RCTs [39, 40, 54, 55], one nonrandomized controlled trial [56] and six pre-post interventional studies or RCTs that are considered as pre-post interventional (i.e., no adequate control group) [57–62].

**Table 2:** Characteristics of included studies, sorted by outcome domain and in descending order according to methodological quality

			Intervention					Outcomes	
**Study / Design**	**Methodo-logical quality**	**Study population gender: (male/female) age: mean ± sd**	**Type**	**Volume**	**Progression**	**Supervision**	**Adherence/attendance**	**Domain**	**Instruments and measures**
Taddei et al. [39] Randomized controlled trial	High	Long distance runners IG (*n* = 14) Gender: 5/9 Age: 41.9 ± 7.4 yr CG (*n* = 14) Gender: 9/5 Age: 41.6 ± 6.0 yr	I: Foot and ankle strength exercise program C: stretching program	8 weeks 4 sessions 20–30 min	Customized, 3 levels (sitting, double leg, single leg stance)	Weekly supervised session	80.4% supervised sessions attended		Barefoot running analysis with 3D motion capture system and force plates:
								Foot and ankle biomechanics	MLA ROM^b^ and stiffness;
								Ground reaction forces	GRF vertical^a^ and AP impulse at push-off.
Okamura et al. [40] Randomized controlled trial	High	Pronated foot posture IG (n = 10) Gender: 1/9 Age: 19.7 ± 0.9 yr CG (*n* = 10) Gender: 2/8 Age: 20.2 ± 1.5 yr	I: Short-foot exercise program supported by electrical stimulation and EMG biofeedback C: No intervention	8 weeks 3 sessions 3 sets 10 reps 5 s contraction 45 s rest between sets	Customized, 3 levels (sitting, double leg, single leg stance)	Initial 20-min training session, weekly supervised session	102.1% unsupervised sessions accomplished; 77.5% supervised sessions attended		Barefoot gait analysis with 3D motion capture system and force plates:
								Foot and ankle biomechanics	Navicular drop^a^ and corresponding moment in time;
								Ground reaction forces	GRF (anterior, medial, vertical^c^) in second half of stance;
								Spatiotemporal parameters	Stance phase duration.
Matsumoto et al. [58] Pre-post intervention study	Low	*n* = 20 Gender: 10/10 Age: 20.0 ± 2.4 yr	I: Short-foot exercise program	4 weeks # sessions NR 30 reps 5 s contraction	Fixed, 3 levels: wk. 1 – sitting wk. 2 – double leg stance wk. 3 to 4 – single leg stance	Initial 30-min training session	67.2% sessions accomplished		Barfoot gait analysis with 3D motion capture system and pressure plate:
								Foot and ankle biomechanics	MLA compression^a^, peak pressure per foot region, total plantar contact area;
								Spatiotemporal parameters	Gait speed.
Lynn et al. [53] Randomized controlled trial	High	IG (n = 8) Gender: 3/5 Age: 23.7 ± 2.1 CG (n = 8) Gender: 3/5 Age: 22.6 ± 1.7 yr	I: Short-foot exercise program C: No intervention	4 weeks daily 100 reps 5 s contraction	Fixed, 2 levels: week 1,2 – sitting week 3,4 – double leg stance	Initial training session, weekly phone consult, extra instructional training session before week 3	NR	Dynamic balance	YBT mediolateral CoP excursion for dominant and non-dominant^c^ stance leg
Lee and Choi [54] Randomized controlled trial	Moderate	Chronic ankle instability IG (n = 15) Gender: 5/10 Age: 20.9 ± 1.1 yr CG (n = 15) Gender: 5/10 Age: 20.8 ± 0.9 yr	I: IFM strength exercise program C: no intervention	6 weeks 3 sessions 3 blocks 4 sets 3–15 reps 3–20s contraction	Fixed, 3 levels:week 1,2 – sitting week 3,4 – double leg stance week 5,6 – single leg stance	Supervision, but not specified	NR	Dynamic balance	YBT composite reach distance normalized to leg length
Mulligan et al. [56] Pre-post intervention study	Moderate	*n* = 21 Gender: 3/18 Age: 26.1 ± 3.7	Short-foot exercise program	4 weeks daily 3 min 5 s contraction	Customized, 3 levels (sitting, double leg, single leg stance) + variations (vision, surface stability)	Initial 1-h training session	85.7% sessions accomplished	Dynamic balance	SEBT reach distance in five directions, among which medial^a^
Tudpor et al. [55] Non-randomized controlled trial	Low	Diabetes IG (*n* = 8) Gender: 4/4 Age: 62.6 ± 0.4 yr CG (*n* = 7) Gender: 3/4 Age: 67.4 ± 0.5 yr	I: foot strength exercises + short-foot exercises C: foot strength exercises	8 weeks daily IG 30 reps of foot exercises + 30 min SFE CG 30 reps of foot exercises	No progression, sitting position	NR	NR	Dynamic balance	SEBT reach distance normalized to leg length in eight directions, among which lateral^a^
Lee et al. [57] Pre-post intervention study	Low	Chronic ankle instability (*n* = 15) Gender: 7/8 Age: 21.5 ± 2.5 yr	Short-foot exercise program	8 weeks 3 sessions 3 sets 12 reps 5 s contraction	2 levels, fixed: week 1 to 4 – sitting weeks 5 to 8 – single leg stance	NR	NR	Dynamic balance	Moving platform: overall and medio-lateral^a^ center of gravity displacement index score
Ma et al. [61] Pre-post intervention study	Low	Chronic ankle instability (n = 14) Gender: 6/8 Age: 20.3 ± 1.5 yr	Short-foot exercise program + sham transcranial direct current stimulation	4 weeks 3 sessions 20 min 4 sets	3 levels (sitting, double leg, single leg stance)	All sessions were supervised	NR	Dynamic balance	YBT composite reach distance normalized to leg length
Kim et al. [59] Pre-post intervention study	Low	Pronated foot function (*n* = 7) Gender: 6/1 Age: 24.0 ± 1.9 yr	Short-foot exercise program	5 weeks 3 sessions 30 min	No progression, sitting position	Initial training session, all sessions were supervised	NR	Dynamic balance	YBT composite reach distance normalized to leg length
Pisal et al. [60] Pre-post intervention study	Low	Pronated foot posture (*n* = 40) Gender: NR Age: NR	Short-foot exercise program	4 weeks daily 100 reps 5 s contraction	NR	NR	NR	Dynamic balance	YBT reach distance of both legs in three directions, among which posterolateral with the right leg^d^

*IG: intervention group, CG: control group, I: intervention, C: control, PIFM: plantar intrinsic foot muscles, MLA: medial longitudinal arch, ROM: range of motion, GRF: ground reaction force, CoP: center of pressure, AP: anteroposterior, CSA: cross-sectional area, US: ultrasound, YBT: Y-balance test, SEBT: star excursion balance test, NR: not reported.*

^*a,b,c,d*^*: indicates the selected outcome measure when multiple measures were reported within the same outcome domain, based on the prioritization: relation with the role of the PIFMs (*^*a*^*), availability of data (*^*b*^*), statistical significance (*^*c*^*), and effect size (*^*d*^*). For example, indication ‘*^*d*^*’ means that several outcome measure were considered equally related to the role of the PIFMs, the data to determine the SMD was available for more than one of these measures and more than one was statistically significant, of which the measure with the largest effect size was selected for further analysis*

The total number of participants was 226 (range: 7–40) with a mean age of 23.3 years. One study included older participants (> 60 yr) and these were diagnosed with diabetes [56]. Other study populations were characterized by chronic ankle instability [55, 58, 62], pronated foot posture [40, 60, 61], or long-distance runners [39]. The three remaining studies [54, 57, 59] included participants without any of such notable common characteristics.

All included studies investigated the effect of a strength exercise program with a duration of four to eight weeks. In nine studies [40, 54, 56–62], the effect of only short foot exercises was investigated. In the two other studies the exercise program was composed of either various intrinsic foot muscle exercises [55] or foot and ankle exercises [39]. The number of prescribed exercise sessions in a week ranged from a minimum of three sessions to daily practice. Half of the programs commenced with an instructional training session [40, 54, 57, 59, 60]. In two studies all sessions were supervised [60, 62]. One out of four weekly sessions was supervised in two other studies [39, 40]. The remainder of the sessions in these and other studies were unsupervised or supervision was not specified. Sometimes a weekly phone consult [54] or an extra instructional training session mid-way was added to the program [54]. The session duration varied from a few to 30 minutes. Most programs were progressive in the level of difficulty, either fixed or customized. Adherence (i.e., the proportion of the prescribed unsupervised sessions that is accomplished) was not reported in six studies [39, 54–56, 58, 61]. In the three other studies that comprised of unsupervised sessions [40, 57, 59], 67.2–102.1% of the prescribed unsupervised sessions were completed. Attendance (i.e., the proportion of the scheduled supervised sessions that is attended) was reported to be 77.5 [40] and 80.4% [39] or was not reported [54, 60, 62].

Dynamic balance performance was the outcome measure in eight studies, using either the star excursion balance test (SEBT) [56, 57], the Y balance test (YBT) [54, 55, 60–62] or a test with a moving platform [58]. Three studies did a gait [40, 59] or running [39] analysis to evaluate the effect of the intervention using various parameters for foot biomechanics [39, 40, 59], GRF [39, 40] and spatiotemporal characteristics [40, 59]. The last column of Table 2 shows the selected outcome measures per domain, based on the predefined prioritization, for further analysis.

### Quality assessment

The results of the quality assessment for ‘reporting’ and ‘internal validity’ are shown in Table 3. The median score for ‘reporting’ was 6 out of 13 (range: 3–11). Three studies [59–61] attained less than half of the points for this category. Almost half of the studies failed to describe the intervention clearly enough and/or lacked to report the losses of patients to follow-up. None of the items for ‘external validity’ (not displayed in Table 3) could be graded due to the lack of detailed information concerning recruitment procedures and the non-medical setting in which the program took place.

**Table 3:** *Quality assessment scores on the items of reporting and internal validity*

	Reporting												Internal validity													
	1	2	3	4	5	6	7	8	9	10	27	Total reporting (max. 12)	14	15	16	17	18	19	20	21	22	23	24	25	26	Total Internal validity (max. 13)
Taddei et al. [39]	1	1	1	1	2	1	1	0	1	1	0	**10**	0	1	1	1	1	1	1	1	1	1	1	1	1	**12**
Okamura et al. [40]	1	1	1	1	2	1	1	0	1	1	1	**11**	0	1	1	1	1	1	1	1	1	1	0	1	1	**11**
Lynn et al. [54]	1	1	1	1	2	1	1	0	1	0	0	**9**	0	0	1	1	1	0^a^	1	1	1	1	0	1	1	**9**
Lee and Choi [55]	1	1	1	0	0	1	1	0	0	1	0	**6**	0	0	1	1	1	0^a^	1	1	1	1	0	0^a^	0^a^	**7**
Mulligan et al. [57]	1	1	1	1	0^b^	1	1	0	1	1	0	**8**	0	0^b^	1	0^b^	1	1	1	0^b^	0^b^	0^b^	0^b^	0^b^	1	**5**
Tudpor et al. [56]	1	1	1	0	1	1	1	0	0	0	0	**6**	0	0	1	1	1	0^a^	0^a^	1	0	0^b^	0^b^	0^a^	0	**4**
Lee et al. [58]	0	1	0	0	0^b^	1	1	0	1	1	1	**6**	0	0^b^	1	0^b^	1	0^a^	1	0^b^	0^b^	0^b^	0^b^	0^b^	1	**4**
Ma et al. [62]	1	1	1	1	0^b^	0	1	1	1	1	1	**9**	0	0^b^	1	0^b^	1	0^a^	1	0^b^	0^b^	0^b^	0^b^	0^b^	1	**4**
Matsumoto et al. [59]	0	1	1	0	0^b^	1	1	0	0	1	0	**5**	0	0^b^	1	0^b^	1	0^a^	1	0^b^	0^b^	0^b^	0^b^	0^b^	0^a^	**3**
Kim et al. [60]	0	1	1	0	0^b^	0	1	0	0	0	0	**3**	0	0^b^	1	0^b^	1	0^a^	1	0^b^	0^b^	0^b^	0^b^	0^b^	0^a^	**3**
Pisal et al. [61]	0	0	0	0	0^b^	1	1	0	0	1	1	**4**	0	0^b^	1	0^b^	0^a^	0^a^	1	0^b^	0^b^	0^b^	0^b^	0^b^	0^a^	**2**

^*a*^*unable to determine.* ^*b*^*not applicable to study design. 1: Objective, 2: Main outcomes, 3: Sample characteristics, 4: Intervention, 5: Confounders, 6: Main findings, 7: Random variability, 8: Adverse events, 9: Lost to follow up, 10: Actual p-values, 27: Sample size calculation, 14: Blinding subjects, 15: Blinding assessors, 16: Data dredging, 17: Different lengths of follow-up, 18: Statistical tests, 19: Compliance, 20: Accurate outcome measures, 21: Same origin of sample, 22: Same recruitment period, 23: Randomization, 24: Concealed assignment, 25: Confounding, 26: Lost to follow up. Studies are presented in descending order according to the score on internal validity*

The total score for the category ‘internal validity’ ranged from 2 to 12. More than half of the studies was classified as being of ‘low’ quality [56, 58–61], leaving two moderate-quality studies [55, 57] and three high-quality RCTs [39, 40, 54] for data synthesis and quality of evidence assessment. The moderate quality studies consisted of one pre-post interventional study [57] that attained the nearly maximum grade for its design and one RCT [55]. Compared to the high-quality RCTs, the moderate-quality RCT [55] did not clearly describe confounders and the loss of subjects to follow-up. Therefore, the raters were unable to determine if items 25 (i.e., adjustment for confounding) en 26 (i.e., accounting for losses to follow-up) were met by the study, resulting in a zero score on these items. Two RCTs of moderate- [55] and high-quality [54] neither described the degree of adherence or attendance nor the measures taken to promote this behavior. The same two studies did not address blinding of the assessors to the allocated intervention. These studies were the only RCTs that assessed dynamic balance as the outcome of the intervention.

### Data synthesis

Five studies were eligible to be included for data synthesis and quality of evidence assessment. Two high quality RCT’s [39, 40] investigated foot function during gait, whereas dynamic balance was the outcome measure in one high-quality RCT [54], one moderate-quality RCT [55] and one moderate-quality pre-post intervention study [57]. The effects of the interventions on the selected outcome measures are presented in Table 4 for studies that were included in the data synthesis (i.e., high- and moderate-quality studies) as well as those not included (i.e., low-quality studies). The effects of the interventions on other outcomes can be found in Additional file 3.

**Table 4:** Intervention effects on foot function during gait and running and dynamic balance

				Intervention group				Control group		Intervention vs. control group		
Study / Design	Methodological quality	Outcome domain	Selected outcome measure	Baseline Mean ± sd	Follow-up Mean ± sd	Within group mean difference	Within group SMD	Baseline Mean ± sd	Follow-up Mean ± sd	Between group difference in change from baseline	Between group SMD in change from baseline ^**c**^	Narrative summary of findings on PIFM strength
Taddei et al. [39] Randomized controlled trial	High	Foot and ankle biomechanics	MLA ROM (°)	4.2 ± 2.4	3.6 ± 2.3	−0.6	0.26	4.6 ± 2.2	4.6 ± 1.8	−0.6	0.26	MRI assessed PIFM volume was significantly increased in IG as opposed to CG, whereas CSA and toe plantar flexion strength remained unchanged
		Ground reaction forces	GRF vertical impulse in second half of stance (N·s)	65.9 ± 7.9	67.9 ± 6.5	2.0	0.28^NR^	74.3 ± 7.0	73.5 ± 6.5	2.8	0.37^†^	
Okamura et al. [40] Randomized controlled trial	High	Foot and ankle biomechanics	Navicular drop (mm)	6.2 ± 1.7	6.2 ± 1.5	0.0	0.00	5.9 ± 2.6	5.4 ± 2.5	0.5	− 0.23	US assessed PIFM thickness in IG and CG remained unchanged
		Ground reaction forces	GRF vertical in second half of stance (% BW)	109.1 ± 4.5	108.3 ± 5.7	−0.8	−0.16	107.5 ± 6.2	108.4 ± 6.5	−1.7	−0.31	
		Spatiotemporal parameters	Stance phase duration (ms)	610.1 ± 36.8	600.4 ± 34.5	−9.7	0.27	623.6 ± 36.8	618.8 ± 47.1	−4.9	0.13	
Matsumoto et al. [58] Pre-post intervention study	Low	Foot and ankle biomechanics	MLA compression (°)	3.72 ± 6.8	3.65 ± 9.8	−0.07	0.01	n/a	n/a	n/a	n/a	Toe grip strength in IG was significantly increased
		Spatiotemporal parameters	Gait speed (m/s)	0.33 ± 0.02	0.33 ± 0.04	0.00	0.00	n/a	n/a	n/a	n/a	
Lynn et al. [53] Randomized controlled trial	High	Dynamic balance	YBT mediolateral CoP excursion for non-dominant stance leg (mm)	52.4 ± 4.5	43.1 ± 5.1	−9.3	1.83*	47.8 ± 7.8	48.1 ± 5.5	−9.6	1.43^†^	n/a
Lee and Choi [54] Randomized controlled trial	Moderate	Dynamic balance	YBT composite reach distance (% leg length)	66.8 ± 9.6	70.9 ± 8.7	6.1^b^	0.66*	65.4 ± 8.7	66.7 ± 9.1	3.8^b^	0.41^†^	n/a
Mulligan et al. [56] Pre-post intervention study	Moderate	Dynamic balance	SEBT reach distance in medial direction (cm)	57.8 ± 7.4	61.6 ± 6.6	3.8	0.54*	n/a	n/a	n/a	n/a	n/a
Tudpor et al. [55] Non-randomized controlled trial	Low	Dynamic balance	SEBT reach distance in the lateral direction (% leg length)	54.8 ± 5.4^a^	53.6 ± 9.6^a^	−1.2	−0.15	59.0 ± 9.5^a^	55.6 ± 6.9^a^	2.2	0.27	n/a
Lee et al. [57] Pre-post intervention study	Low	Dynamic balance	Medio-lateral center of gravity displacement index score as a response to a moving platform	3.4 ± 1.0	1.5 ± 0.8	−1.9	1.98^NR^	n/a	n/a	n/a	n/a	n/a
Ma et al. [61] Pre-post intervention study	Low	Dynamic balance	YBT composite reach distance (% leg length)	97.0^d^ ± 7.5^a,d^	96.0^d^ ± 7.5^a,d^	−1.0	−0.13	n/a	n/a	n/a	n/a	n/a
Kim et al. [59] Pre-post intervention study	Low	Dynamic balance	YBT composite reach distance (% leg length)	74.3 ± 8.3	82.4 ± 7.4	8.1	0.97*	n/a	n/a	n/a	n/a	n/a
Pisal et al. [60] Pre-post intervention study	Low	Dynamic balance	YBT reach distance of right leg in posterolateral direction	61.1 ± 5.2	65.1 ± 5.1	4.0	0.78*	n/a	n/a	n/a	n/a	n/a

*Studies are sorted by outcome domain and in descending order according to methodological quality*

† significant group x time interaction effect, * significant effect for time in the intervention group, ^*NR*^ *significance not reported*

*sd: standard deviation, SMD: standardized mean difference, PIFM: plantar intrinsic foot muscles, MLA: medial longitudinal arch, ROM: range of motion, CoP: center of pressure, IG: intervention group, CG: control group, CSA: cross-sectional area, GRF: ground reaction force, BW: body weight, US: ultrasound, YBT: Y-balance test, SEBT: star excursion balance test*

^*a*^ sd derived from the reported standard error of the mean (SEM) according to the formula: SEM * $\sqrt{n}$*,* ^*b*^ *value adopted form the article,* ^*c*^ *positive values indicate an improvement in the outcome measure favoring the intervention and vice versa,* ^*d*^ *value estimated from graph*

#### Foot function during gait and running

The effect of PIFM strengthening exercises on foot function during gait and running was investigated by a an 8-week short-foot exercise program in individuals with a pronated foot posture [40] and by a foot and ankle exercise program of the same duration in younger to middle-aged long-distance runners [39].

The results on foot and ankle biomechanics were limited to the effect on MLA motion. Non-significant changes were found in either the navicular drop during gait in healthy young adults with pronated foot posture (SMD: − 0.23) [40] or the range of motion in the mid foot joint during running in long-distance runners (SMD: 0.26) [39]. Consequently, there is no evidence supporting the effect of PIFM strengthening exercises on MLA kinematics.

Deviating results were found concerning the effect of PIFM strengthening exercises on GRF in the late stance phase. Vertical GRF impulse during running push-off was significantly increased in long-distance runners that participated in a foot and ankle exercise program (SMD: 0.37) [39], whereas vertical peak GRF during gait remained unchanged in younger adults with pronated foot posture that were involved in a short-foot exercise program (SMD: − 0.31) [40]. The beneficial effect that was found for the foot and ankle exercise intervention was accompanied by an increase in PIFM volume [39]. The certainty of evidence for the effect of PIFM strengthening exercises on vertical GRF was graded ‘low’ due to the inconsistency in findings and the imprecision of the data (i.e., small sample sizes).

Stance phase duration of gait was the only spatiotemporal parameter eligible for the synthesis and investigated by one study [40]. No effect of the short-foot exercise program was found on this outcome measure (SMD: 0.13) [40].

#### Dynamic balance control

Both studies that investigated the effect of a 4-week short foot exercise program [54, 57] as well as the study in which participants were enrolled in a more comprehensive 6-week PIFM exercise program [55] showed a significant improvement in performance on a dynamic balance test in the intervention group (SMD: 0.54–1.83) that was not present in the control group of the RCTs [54, 55]. When the statistical non-significant change from baseline on balance performance of the control group was taken into account, the controlled trials [54, 55] further demonstrated an SMD of 0.41 for composite reach distance on the Y-balance test in individuals with chronic ankle instability [55] and an SMD of 1.43 for mediolateral displacement of the CoP while performing the SEBT in healthy young adults [54]. These SMDs of the change from baseline between the groups was 9% [55] and 18% [54] lower than the within group SMD of the intervention group for the same studies.

Based on the finding that the one high-quality study [54] also had some methodological shortcomings (e.g., no description of adherence/attendance and blinding of assessor) that led to concerns on the risk of bias, the certainty of evidence was downgraded by two levels. Due to the imprecision of the results (i.e., small sample sizes), the level of evidence for the effect of PIFM strengthening intervention on improving dynamic balance was further downgraded to a final grade of ‘very low’.

The sensitivity analyses only pertained to studies on the outcome domain of dynamic balance. The level of evidence was not affected by a more progressive or conservative cut-off value for moderate-quality studies.

## Discussion

The aim of the study was to provide insight in the evidence for the effect of interventions anticipated to improve PIFM strength on dynamic balance control and foot function during gait in adults. Only five studies with small sample sizes were of sufficient methodological quality to be included for data synthesis. This indicates that little is known about the effect of PIFM strengthening interventions on fall-related dynamic function. The results were limited to the effects of only strength training interventions in a primarily younger population. Low-certainty evidence revealed the beneficial effect of PIFM strengthening exercises on vertical GRF. Very low-certainty evidence showed that PIFM strength training improved dynamic balance control. Additionally, there was a lack of evidence for the effect of PIFM strengthening exercises on MLA kinematics.

The low-certainty evidence for the beneficial effect of PIFM strengthening exercises on vertical GRF impulse was based on the small improvement of this parameter during running as a result of the intervention in a high-quality proof-of-principle RCT. This improvement was not only accompanied, but also associated, with hypertrophy of the PIFMs [39]. Therefore, the effect could be carefully ascribed specifically to the PIFM exercises as part of the comprehensive foot and ankle exercise program. PIFMs are known to facilitate in stiffening the foot during late stance enabling efficient force transmission [26], which could be the mechanisms supporting the finding on increased vertical GRF impulse in healthy long distance runners [39]. Although the authors did not clarify if the enhanced impulse was the result of increased vertical GRF or an unfavorable prolonged push-off phase, they interpreted the enhanced impulse as less energy consuming and therefore increased amount of GRF is more plausible.

For dynamic balance control, very low-certainty evidence showed improvement after a PIFM strengthening program. Although the diverse studies consistently demonstrated an improvement after the intervention, there were not only major concerns on the risk of bias, but also on the instruments and the measures that were used. It is remarkable that a large effect (SMD: 1.43) was only demonstrated by the study with the least concern on all of these aspects [54]. This high-quality study assessed the mediolateral direction of balance, which is most relevant from the perspective of the PIFMs. This was also the only study that used highly accurate instrumented equipment (i.e., a force plate) to assess balance during the performance of a leg reach test. In contrast, the other two studies [55, 57] manually measured the distance reached by the leg which is merely the result of a movement measured at one instance in time, rather than being a measure of balance control while reaching [63]. This makes it is disputable how the reach performance on a dynamic balance test (e.g. SEBT, YBT), which is also predominantly applicable to physically active individuals [64], relates to balance control during gait [65] which is the topic of interest when it comes to the risk of falling.

Whilst the studies that evaluated balance as the outcome of the intervention all applied isolated PIFM exercises (e.g., short-foot exercise), none of them additionally assessed PIFM strength. This implies that the improvement was not the result of stronger extrinsic foot muscles, nor can it be ascribed to stronger intrinsic foot muscles with certainty. Other mechanisms than strength gains could have mediated the intervention effect, such as improved neuromuscular control [55], proprioception, or plantar sensation [66]. A learning effect also could have occurred as the SMDs of the changes from baseline between groups were a maximum of 18% smaller than the within group SMDs of the same studies [54, 55].

The lack of evidence for the effect of PIFM strengthening exercises on MLA kinematics may be explained by the study population in combination with the investigated activities (i.e., gait [40, 59] and running [39]). Firstly, there was no indication of abnormal dynamic MLA motion in the selected samples. This also applies to the study of Okamura et al. [40] including participants with pes planus alignment, as a statically assessed foot posture does not correlate well with the dynamic behavior of the MLA [67]. Secondly, the PIFMs seem to contribute only marginally to MLA motion control during loading in gait and running in healthy younger adults [26]. Both of these explanations may have mitigated the effect of the observed gain in toe plantar flexion force [59] or PIFM force capacity [39] on MLA motion.

Only one study that met our selection criteria [56] was characterized by a study sample consisting of older adults. However, due to the poor methodological quality it was not included in the synthesis. We revisited the excluded records and found that the limited number of eligible studies in older adults could not be attributed to the criterion to exclude studies in participants presented with pain. The scarcity of studies in older adults is remarkable as this population is known to have diminished PIFM force producing capacity [8] and concomitant gait deficiencies that are related to the role of the PIFMs. Older adults seem to be as responsive to PIFM strength training as younger adults with respect to neural and muscular adaptation [30], but they may potentially benefit more with respect to functional improvements. Whereas younger adults may not use the full capacity of PIFM strength during gait, older adults’ gait may be more demanding. Stronger PIFMs could then sooner result in a corresponding improvement of their gait, which is expected to decrease the risk of falling [5]. Therefore, studies with older adults are merited to build on the evidence for the effect of PIFM strengthening exercises on dynamic function in this specific population. Based on the forgoing discussion on the body of evidence, these studies should be of high quality and are recommended to assess meaningful fall-related parameters such as balance capabilities during gait and propulsive force generation.

Strength training for older adults targeting the PIFMs involves some additional aspects that need to be taken into account when investigating interventions in this specific population. First, plantar cutaneous somatosensation and proprioception, predominantly in the distal joints, declines with aging [68]. As a consequence, decreased awareness of plantar loading and diminished joint position and motion sense may be encountered. This may encumber the ability to properly execute the required exercise movements and therefore may hamper the effectiveness of training. Second, the motor control of primarily complex tasks is affected in older adults [30]. PIFM exercises are complex in nature, illustrated by the learning curve in motor performance of the task [69] and the inability to perform these exercises even by younger adults [70]. Both the diminished afferent information and reduced motor control in combination with the complex nature of PIFM exercises underpin the disputability of purely PIFM training for strengthening the PIFMs in older adults. Enhancing sensory afferent information [70] and providing biofeedback from muscle activity and plantar pressure [41, 71] have been suggested for PIFM training to overcome these deficits. However, the execution of PIFM exercises (e.g., the short-foot exercise) requires a voluntary contraction of the PIFMs that is very dissimilar to everyday activities such as walking, adding another challenge regarding the suitability of PIFM exercises aiming to improve gait in older adults.

The one study with a significant beneficial effect on gait function [39] consisted of a comprehensive foot and ankle exercise program including heel raising. Such exercises are characteristic for balance and functional training that has been advocated to be the primary kind of training within fall prevention interventions, rather than only resistance training [72]. Indeed, heel raising is a common aspect of fall prevention programs [73–75]. Moreover, older adults who participated in a multifaceted podiatry intervention exhibited a reduced number of falls, and this was predominantly attributed to the foot and ankle exercises [35]. Primarily intended to strengthen the ankle plantar flexor muscles [76], raising the heel off the ground also requires the foot to act as a rigid lever. This may require PIFMs to be active in a similar way as compared to when they contribute to foot stiffening for push-off during gait [18, 26]. Although common within fall preventions programs, only few studies [32, 39, 77] investigated the effect of foot and ankle strengthening exercises on fall-related dynamic balance and gait parameters and one study [39] assessed a measure of PIFM strength simultaneously. In order to better understand the role of the PIFMs in the benefits of functional foot and ankle exercises as components of fall prevention programs and to formulate related recommendations, future studies should evaluate changes in PIFM strength or strength capacity next to outcome measures related to dynamic foot function and balance control.

Several limitations of the study need to be taken into account. Most importantly, there was heterogeneity in study populations, interventions and the investigated activities within outcome domains (e.g. walking vs. running). The extent to which studies were similar enough to be grouped together may be questionable. Therefore, the results of this study must be interpreted with caution. However, the diversity in study populations is not expected to have confounded the results to a large extent. The execution of and adherence to the exercises, and thus the intervention effects, is not likely to be influenced by the characteristics of most study populations (e.g., pronated foot posture). On the other hand, it is questionable how this applies to a population of individuals with chronic ankle instability. The one study with this population that was included in the synthesis still showed a significant intervention effect [55]. Regarding the variety of activities of interest, the single significant effect of PIFM strengthening exercises on vertical GRF impulse was found for running only [39]. It remains elusive how this can be generalized to walking due to the limited existence of similar studies for walking. Another limitation is that a meta-analysis was not possible due to the unknown variance of changes from baseline for the majority of studies. Therefore, the effect estimates were summarized rather than providing a combined estimate of the average treatment effect. The accompanying drawback of this method is that it does not account for the differences in sample sizes across the studies. This only applied to the outcome domain of dynamic balance, since the sample size of the respective studies varied. Lastly, the pooled pre-intervention standard deviation was chosen as an alternative for the denominator in the SMD calculation of the changes from baseline. However, it is expected that this standard deviation is smaller than that of the change from baseline, as was apparent in the study of Lee and Choi [55]. Therefore, it is likely that the reported SMDs underestimated the true SMDs.

## Conclusion

This review revealed at best low-certainty evidence that PIFM strengthening exercises improve foot function during gait and very low-certainty evidence for its favorable effect on dynamic balance control. This was based on the findings from a limited number of high-quality studies with small samples of primarily healthy younger adults. In order to build on the body of evidence for strengthening the PIFMs from the perspective of the prevention of falling, there is a need for high-quality studies that aim to investigate the effect of functional strengthening exercises targeting the PIFMs in large samples of older adults. The outcome measures should be related to either fall risk and the role of the PIFMs such as propulsive forces or balance during walking in conjunction with PIFM strength measures.

## Supplementary Information

**Additional file 1.** Search strategies for each database.

**Additional file 2.** Modified Downs & Black checklist as it was used to assess the quality of the studies included in the review.

**Additional file 3.** Data set and data analysis upon which the reported findings are based.

## Abbreviations

PIFM: Plantar intrinsic foot muscle
CoP: Center of pressure
MLA: Medial longitudinal foot arch
RCT: Randomized controlled trial
GRF: Ground reaction force
SMD: Standardized mean difference
diff: Difference
SEBT: Star excursion balance test
YBT: Y balance test
