# Supplementary material for: The effect of interventions anticipated to improve plantar intrinsic foot muscle strength on fall-related dynamic function in adults: a systematic review
Source: J Foot Ankle Res. 2022 Jan 20;15:3. doi: 10.1186/s13047-021-00509-0 (PMC8772142; doi:10.1186/s13047-021-00509-0)
Supplement: Supplementary file 1 — Additional file 1. Search strategies for each database. [file 13047_2021_509_MOESM1_ESM.docx]

### Search strategies

**Pubmed**

#1 “exercise therapy” [MeSH Terms] OR “resistance training” [MeSH Terms] OR exercise OR strengthening OR shoes OR footwear OR barefoot OR foot ortho* OR insole* OR inlay*

#2 doming OR “short foot” OR “foot core” OR foot musc* OR intrinsic foot OR plantar musc* OR toe musc* OR hallu* muscle

#3 postur* balance OR postur* stability OR postur* control OR stance balance OR stance stability OR stance control OR dynamic* OR function* OR gait OR walking OR locomotion OR running

#4 #1 AND #2 AND #3

#5 stroke OR “multiple sclerosis” OR “cerebral palsy”

#6 #4 NOT #5, “Humans, Dutch, English, from 2010-2020”

**CINAHL Plus with Full Text (EBSCOhost interface)**

#1 MH “Muscle Strengthening+” OR exercise OR strengthening OR shoes OR footwear OR barefoot OR foot ortho* OR insole* OR inlay*

#2 doming OR “short foot” OR “foot core” OR foot musc* OR intrinsic foot OR plantar musc* OR toe musc* OR hallu* muscle

#3 postur* balance OR postur* stability OR postur* control OR stance balance OR stance stability OR stance control OR dynamic* OR function* OR gait OR walking OR locomotion OR running

#4 #1 AND #2 AND #3

#5 stroke OR “multiple sclerosis” OR “cerebral palsy”

#6 #4 NOT #5, Limiters - Published Date: 20100101-20200731; Peer Reviewed; English Language; Expanders – Apply related words; Also search within the full text of the articles; Apply equivalent subjects Search modes - Boolean/Phrase

**SPORTDiscus with Full Text (EBSCOhost interface)**

#1 DE “STRENGTH training” OR DE “EXERCISE therapy” OR DE “RESISTANCE training” OR exercise OR strengthening OR shoes OR footwear OR barefoot OR foot ortho* OR insole* OR inlay*

#2 doming OR “short foot” OR “foot core” OR foot musc* OR intrinsic foot OR plantar musc* OR toe musc* OR hallu* muscle

#3 postur* balance OR postur* stability OR postur* control OR stance balance OR stance stability OR stance control OR dynamic* OR function* OR gait OR walking OR locomotion OR running

#4 #1 AND #2 AND #3

#5 stroke OR “multiple sclerosis” OR “cerebral palsy”

#6 #4 NOT #5, Limiters - Published Date: 20100101-20200731; Peer Reviewed; Language: English; Publication Type: Academic Journal; Document Type: Article, Expanders – Apply related words; Also search within the full text of the articles; Apply equivalent subjects Search modes - Boolean/Phrase

**PEDro**

Abstract & Title: foot

Therapy: strength training; orthoses, taping, splinting

Body Part: foot or ankle

Subdiscipline: musculoskeletal

**Web of Science**

#1 TS=“resistance training” OR TS=exercise OR TS=strengthening OR TS=shoes OR TS=footwear OR TS=barefoot OR TS=foot ortho* OR TS=insole* OR TS=inlay*

#2 TS=doming OR TS= “short foot” OR TS= “foot core” OR TS=foot musc* OR TS=intrinsic foot OR TS=plantar musc* OR TS=toe musc* OR TS=hallu* muscle

#3 TS=postur* balance OR TS=postur* stability OR TS=postur* control OR TS=stance balance OR TS=stance stability OR TS=stance control OR TS=dynamic* OR TS=function* OR TS=gait OR TS=walking OR TS=locomotion OR TS=running

#4 #1 AND #2 AND #3

#5 TS=stroke OR TS= “multiple sclerosis” OR TS= “cerebral palsy”

#6 #4 NOT #5

#7 #6 *AND* **LANGUAGE**: (English OR Dutch) *AND* **DOCUMENT TYPES**: (Article)
